# Effect of Polymethylmethacrylate-Hydroxyapatite Composites on Callus Formation and Compressive Strength in Goat Vertebral Body

**DOI:** 10.5704/MOJ.1811.002

**Published:** 2018-11

**Authors:** IS Komang-Agung, L Hydravianto, O Sindrawati, PS William

**Affiliations:** Department of Orthopaedics, Airlangga University, Surabaya, Indonesia; *Department of Pathology, Widya Mandala Katholic University, Surabaya, Indonesia; **Emergency Room Department, Jombang General Hospital, Jombang, Indonesia

**Keywords:** hydroxyapatite, polymethylmethacrylate, vertebral compression fracture, vertebroplasty

## Abstract

**Introduction:** Percutaneous vertebroplasty (PV) is one of the available treatments for vertebral compression fracture (VCF). Polymethylmethacrylate (PMMA) is the most common bone substitute used in the procedure, but it has several disadvantages. Bioceramic material, such as hydroxyapatite (HA), has better biological activity compared to PMMA. The aim of this study was to find an optimal biomaterial compound which offers the best mechanical and biological properties to be used in PV.

**Materials and Methods:** This was an experimental study with goat *(Capra aegagrus hircus)* as an animal model. The animals’ vertebral columns were injected with PMMA-HA compound. Animal samples were divided into four groups, and each group received a different proportion of PMMA:HA compound. The mechanical and biological effects of the compound on the bone were then analysed. The mechanical effect was assessed by measuring the vertebral body’s compressive strength. Meanwhile, the biological effect was assessed by analysing the callus formation in the vertebral body.

**Results:** The optimal callus formation and compressive strength was observed in the group receiving PMMA:HA with a 1:2 ratio.

**Conclusion:** A mixture of PMMA and HA increases the quality of callus formation and the material’s compressive strength. The optimum ratio of PMMA:HA in the compound is 1:2.

## Introduction

In the United States, more than 700,000 cases of vertebral compression fracture (VCF) occur each year, of which 150,000 require hospital admission. Percutaneous vertebroplasty (PV) is one of the available treatments for VCF when conservative treatment is not possible or fail to produce satisfactory results^1^. PV is a minimally invasive imaging-guided technique involving injection of biomaterial into the fractured vertebral body. The advantages of this procedure are: fracture stabilisation, significant long-lasting pain relief and improvement of patients’ mobility, with only a small risk of complications^2^. The most common biomaterial used in this procedure is polymethylmethacrylate (PMMA). Although it has considerable compressive strength, PMMA has some disadvantages: it is non-bioactive, non-unifiable with the hosts’ bone, impermeable by growth factors and chemotherapeutic agents, non-osteoconductive, and biologically incompatible to its environment. In addition, PMMA is unable to stimulate the biological material necessary for bone formation. It is also non-biodegradable, and has the potential to cause severe hypotension due to the release of its monomer during its use in the procedure. Exothermic reaction which occurs during its setting could also cause bone necrosis^3^.

Currently, porous calcium ceramics have proven to be a biocompatible bone substitute. The most common used bioceramic based on calcium phosphate is hydroxyapatite (HA). Its chemical composition, Ca10(PO4)6(OH)2, is the most stable state of calcium phosphate^4^. The biomaterial HA is known to have biocompatible, bioactive, inert, biodegradable, osteoconductive, and osteophilic properties. In some studies, HA has shown the ability to stimulate osteointegration, growth of bone-forming cells (osteoblasts), collagenisation of the surface area of the host bone, and callus formation in the first trimester of postnatal life. An 18-74% new bone growth is observed after HA is implanted into a bone defect^5^. There are several studies which have studied the combination of these two materials, with or without other material^6,7^.

Based on the advantages and disadvantages of HA and PMMA as a biomaterial, our study aimed to examine the effectiveness of PMMA-HA composites in vertebroplasty procedures, both mechanically and biologically. The mechanical strength measured was the compressive strength of the vertebral body after injection of PMMA-HA composites. The biological effectiveness measured was the degree of callus formation. In addition, we assessed the optimal ratio of PMMA and HA in the composite and hoped to achieve the optimal biomaterial combination with mechanical and biological advantages.

Through this study, we hoped to offer a new perspective to physicians, especially surgeons who are familiar with biomaterial use, and spine surgeons, and that the findings in this study could be applied clinically to improve the care of patients with VCF.

## Materials and Methods

This study is an experimental study using goat *(Capra aegagrus hircus)* as an animal model. All procedures regarding the animal samples received ethical clearance from the Animal Care and Use Committee of Veterinary Medicine Faculty of our University. The vertebroplasty procedure involved implantation of either PMMA or PMMA-HA composites into the vertebral body of goat.

This prospective study used a post-test with control group design. The animals were humanely put down by the end of the third week to evaluate the vertebral body both in the experimental and control groups. The evaluation was performed at the end of the third week as by then osteogenesis and bone formation had allowed sufficient callus formation and compression strength to be evaluated.

The samples used in this study were goat vertebral segments and the animals were provided by a local herder. All animals were healthy males, aged 9-12 months with a weight of 21-22 kg. The exclusion criteria were defect or trauma to the associated vertebral segment during the study period, infection of the vertebral segment and death of the animal during the study period. The animals were first housed and acclimatised for seven days the veterinary hospital prior to the intervention. All animals were kept in cages of similar dimension for 21 days after the intervention and they were fed with the same food.

Goat, though a quadruped animal, may be used as animal model in this study because the goats’ vertebral body is mainly loaded in its axis like humans’. The problem with using goats’ vertebral body is that they have relatively stronger compressive strength than humans^8^. The bone remodelling rate in goat is nearly identical to human^9^.

The calculation using sample size formula showed that 32 vertebral segments were required. There were four animal samples and eight vertebral segments were used from each animal. The segments used in this study were the twelfth to thirteenth thoracic vertebrae, and first to sixth lumbar vertebrae (Th 12, Th 13, L1-L6). All segments were considered homogenous.

Biomaterials used in this study were PMMA and HA, both in powder form. The PMMA used was DePuy CMW 3 [DePuy International Ltd, Leeds, USA]. The HA used was synthetic hydroxyapatite, of Osteo-G brand [Central Medical Technologies Inc, Taipei, Taiwan] and was designed to be mixed with PMMA. Equipment used included the cage for the sample sized 6m x 4m x 2m, basic surgical set, orthopaedic surgical set, object glasses, cover glasses, microscope, micrometre and the Shimadzu Autograph Universal Testing Machine series AG-100k NE (10 TE). The husbandry and intervention of the animals were carried out in the Unified Basic Science Laboratory of the veterinary hospital. The mechanical strength of the vertebral bodies was tested in the same laboratory. Histopathological examinations were performed in the Pathology Laboratory of an oncology hospital in the region.

All animals which met the inclusion criteria were anaesthetised before vertebroplasty. The animals were classified according to four treatments. Each group of vertebrae was injected with PMMA-HA composites with different ratios as follows: Control (C)= PMMA only without HA; O1= PMMA:HA (1:1) = 1g:1g; O2= PMMA:HA (1:2) = 1g:2g; O3= PMMA:HA (1:3) = 1g:3g.

The animals’ vertebral columns were exposed using aseptic technique and under general anaesthesia. The injection’s entry point was identified as the conjunction between the horizontal axis of the transverse process and the inferolateral border of the facet joint. The entry point was drilled using a 2mm drill transpedicular to the posterior side trough pedicle cortex. The diameter of the hole may be increased to 3mm. The hole was as deep as the length of the pedicle (±4cm). The amount of biomaterial injected was 2ml in each segment. The material was left in for 15 minutes for proper setting.

After the procedure was finished, the operating area was washed with normal saline and the wound was closed. The animals received postoperative analgesics and antibiotics for three days. Wound care began three days after surgery and continued each day until the wound was healed. The skin suture was removed on Day 10.

The intervention group was injected with increasing amounts of HA because the main concern with PMMA use is its poor bone formation around the biomaterial. We hoped that the osteoconductive properties of HA would give better new bone formation around the biomaterial. The control group represented the result of conventional vertebroplasty, in which only PMMA was used. No group received biomaterial only containing HA, because HA alone was not mechanically strong enough to support the vertebral body.

The animals were killed at the end of the third week to evaluate the vertebral segments. The mechanical strength of the samples was tested in our laboratory. Compressive force was applied to the vertebral segment along its longitudinal axis using the Shimadzu Autograph Universal Testing Machine Series AG-100 k NE (10TE). The force applied was increased gradually until the vertebral body collapsed.

After mechanical testing, samples were preserved with 10% buffered formaldehyde solution, then labelled and sent to the Pathology Laboratory of an oncology hospital in the region. Callus quality was assessed histologically. The quality was evaluated by the presence of osteogenesis, primitive mesenchymal cells (osteoblast progenitors) and osteoblasts. The presence and amount of the callus formed, together with the presence and degree of endochondral ossification, were also evaluated. The quality of the callus was graded based on the ratio of components of cartilage or fibrous tissue to the total callus area. The total area was calculated using graticule grid. The callus quality was graded as follows; 0: Absence of callus formation; +1: Minimal callus formation, defined as 10% of the total area of the callus occupied by cartilage/fibrous tissues, with primitive mesenchymal cells; +2: Moderate callus formation, defined as 10%-25% of the total area of the callus occupied by cartilage/fibrous tissues, and the presence of endochondral ossification; +3: Maximum callus formation, defined as 25%-40% of the total area of callus or more occupied by cartilage/fibrous tissues, and the presence of endochondral ossification.

Quantitative results were presented as mean ± SD. Statistical analysis was performed using one-way ANOVA to compare compressive strength between groups. The least significant difference (LSD) test was used to find out which group had the strongest compressive strength. The comparison of callus formed among all groups was analysed using the Kruskal-Wallis test. In addition, the Mann-Whitney U test was used to compare callus formation between each group.

## Results

The thirty-two vertebral segments were examined after three weeks. The results from the C group and three intervention groups (O1, O2, O3) presented callus formation. The callus formation was highest in group O2, and the Kruskal-Wallis test found significant differences in callus formation among all groups (p<0.001). In addition, the Mann-Whitney U test showed there was a significant difference between each group, except between group O1 and O3 (Table I).

**Table I:** Callus formation in each group

Group	Callus Formation (%)				
	0 (%)	+1 (%)	+2 (%)	+3 (%)	n
Control	8 (100%)				8 (100%)
O1	5 (62.5%)	3 (37.5%)			8 (100%)
O2	1 (12.5%)		4 (50%)	3 (37.5%)	8 (100%)
O3	2 (25%)	4 (50%)	2 (25%)		8 (100%)

Kruskal-Wallis test found significant difference in callus formation among all groups (p<0.001). The test also shows that group O2 (PMMA: HA= 1: 2) has the highest mean rank among all groups

**Fig. 1:**
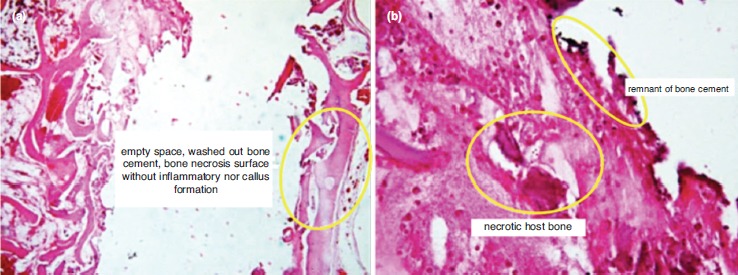
Histology features of the control group (100% PMMA); (a) Empty space from washed out PMMA; the host bone showing absence of callus formation or inflammatory reaction (5X, HE). (b) Remnant PMMA material along the empty space; host osteonecrosis due to heat of the cement material (20X, HE).

**Fig. 2:**
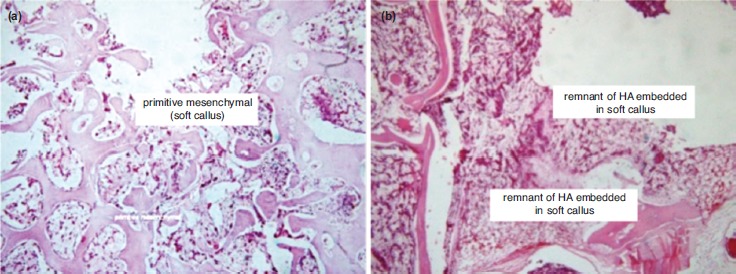
Histology features of group O1 (HA: PMMA ratio 1:1); (a) The space was partially filled with callus formation (20X, HE). (b) The callus embedded in the remnant of HA material (20X, HE).

**Fig. 3:**
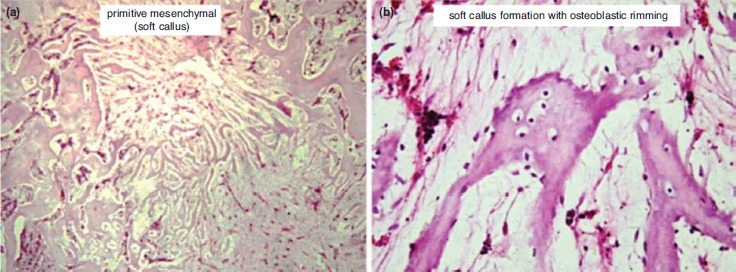
Histology features of Group O2 (HA:PMMA ratio 2:1); (a) The space was entirely filled with callus formation (5X, HE). (b) The callus showed calcified trabecule, minimal remnant of HA material (20X, HE).

**Fig. 4:**
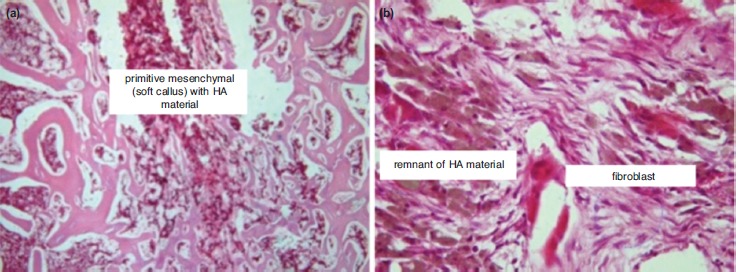
Histology features of Group O3 (HA:PMMA ratio 3:1); (a) The space gap was partially filled with callus formation (5X, HE). (b) A prominent remnant of HA material in fibrous callus formation (20X, HE).

Histopathologic examination using HE revealed empty space from washed out PMMA in C group. The host bone showed neither callus formation nor inflammatory reaction with remnant PMMA material along the empty space, thus showing a host osteonecrosis due to heat from the PMMA material (Fig. 1). In Group O1, the space was partially filled with callus formation. The callus was embedded in the remnant of HA material (Fig. 2). In Group O2, the space was entirely filled with callus. The callus showed calcified trabecule and minimal remnant of HA material (Fig. 3). Meanwhile in Group O3, the space gap was partially filled with callus formation with prominent remnant of HA material among fibrous callus formation (Fig. 4).

The highest compressive strength is shown in O2 groups, followed by Groups O3 and O1 respectively (Table II). Statistical analysis using one-way ANOVA test showed a significant difference between the average compressive strength of groups with different PMMA-HA ratios (p<0.001). A post-hoc test using least significant difference (LSD) showed a significant difference (p<0.05) between all groups (Table III).

**Table II:** Average compressive strength in each group

	n	Mean	Standard Deviation
Control	8	1450 N	30.24
O1	8	1560 N	103.65
O2	8	1840 N	70.91
O3	8	1750 N	69.08
Total	32	1650 N	170.75

**Table III:** Comparative result between each O1, O2, O3, C

Group	Control	O1	O2	O3
Control		0.063	0.000*	0.003*
O1	0.063		0.001*	0.084
O2	0.000*	0.001*		0.008*
O3	0.003*	0.084	0.008*	

Two-way ANOVA was used to assess the difference of compressive strength between different vertebral segments derived from the animals. There was no significant difference between different vertebral segments, but there was a significant difference between groups. A post-hoc test using LSD only showed a significant difference in the sixth lumbar segment if compared with the twelfth and thirteenth thoracic segments and the fifth lumbar segments (p<0.05).

## Discussion

This study shows that optimal callus formation was observed in Group O2, with PMMA:HA ratio of 1:2. In addition, the quality of the callus in the control group was poor. There was no callus formation observed in the control group. In some samples, bone necrosis was observed. This is understandable as PMMA has a high exothermic temperature which may impede callus formation^10^. Moreover, the physical properties of PMMA may lead to bone necrosis and mechanical failure^11^. On the other hand, the biological properties of HA allow bone formation up to one month of healing and HA has excellent bioactivity and osteoconductivity^12, 13^.

The improved callus formation in Group O2 compared to Group O3 was probably caused by an incomplete HA reaction in Group O3 within three weeks. The presence of giant cells in Group O3 also showed that the material was still considered as foreign material which induced an immunological reaction. If observation were to be continued, the process could subside and more bone formation possibly occur. The giant cell itself cannot induce bone resorption but recent study shows the giant cell dissolves HA^14^. Biodegradation is one of the disadvantages of calcium-phosphate-based cement. If the material undergoes degradation before new bone formation occurs, the vertebral body strength will be compromised^15^.

The advantage observed in Group O2 is probably caused by better surface contact. HA has a high degree of porosity^16, 17^. In Group O3, in which there was larger proportion of HA, the porosity of the compound material would decrease its density which in turn led to less contact surface. Less contact surface inhibited bone formation in the implantation site. Also, HA provided direct bone contact with the implant material, thus facilitating bone formation. Meanwhile, fibrous tissue often surrounded the implant when PMMA only was used^18^.

The presence of callus and osteoblasts in the compound group (PMMA-HA) can be explained by the ability of HA to reduce PMMA’s exothermic temperature during the setting process^10^. Previous studies have found the compound had decreased setting temperature from 111^0^ C to 87^0^ C. Additionally, the compound did not decrease setting time, thus minimising bone necrosis^19^. Another advantage of HA was that the material increases the hydrophilic properties of the compound which in turn increased cellular growth and proliferation^20^.

The quantitative assessment of the vertebral segments’ compressive strength showed a significant difference between the four groups (p<0.05). Group O2 was found to have the best compressive strength. This suggested that the compressive strength was positively correlated to callus formation. The group which received PMMA only also had excellent compressive strength. However, the presence of callus and bone deposition in groups receiving the PMMA-HA compound yielded better bone strength. A German study found that PMMA induced local tissue toxicity which could promote fibrous tissue formation. This fibrous tissue may decrease the overall compressive strength of the vertebral body^21^. Another study found that the addition of 2.5% by weight of HA to PMMA yielded superior compressive strength. However, the same study found that addition of more than 2.5% by weight would eventually decrease the compressive strength^22^.

HA as bone substitute is recommended in vertebroplasty due to its osteoconductive property and its similarity to bone mineral. In addition, HA has a high affinity for angiogenic cytokines such as VEGF (Vascular Endothelial Growth Factor)^23^. However, due to its mechanical weakness and its unpredictability, several types of composite such as the one used in this study could be used as an alternative^15, 24^.

The result of this study is in accordance with other reports. A study by Aghyarian *et al* found that calcium-phosphate-containing cement has desirable biological activity without sacrificing mechanical strength^6^.

There are some limitations in this study. Compressive strength was only one of the indicators for assessing the strength of bone substitute material. Other factors such as shear strength, stability during bending, or cement protrusion should be included in future studies. Future studies also may use goats as osteopenic animal model. A study in China shows that goats can be developed as osteopenic animal models^25^. This is important as most compression fractures happen in elderly osteoporotic patients. Moreover, a longer study time is necessary to draw a more complete conclusion in future studies.

## Conclusion

The mixture of PMMA and HA increases the quality of callus formation and the material’s compressive strength. The optimal ratio of PMMA:HA in the compound is 1:2. This ratio leads to the best callus quality and compressive strength compared to the others.

## Conflict of Interest

The authors declare no conflict of interest. This research received no specific grant from any funding agency in the public, commercial or not-for-profit sectors and also no financial support for the research, authorship, and/or publication of this article.
